# Reconciling the regulatory role of Munc18 proteins in SNARE-complex assembly

**DOI:** 10.1107/S2052252514020727

**Published:** 2014-10-21

**Authors:** Asma Rehman, Julia K. Archbold, Shu-Hong Hu, Suzanne J. Norwood, Brett M. Collins, Jennifer L. Martin

**Affiliations:** a Division of Chemistry and Structural Biology, Institute for Molecular Bioscience, University of Queensland, St Lucia, QLD 4072, Australia; b Division of Molecular Cell Biology, Institute for Molecular Bioscience, University of Queensland, St Lucia, QLD 4072, Australia

**Keywords:** SM proteins, SNARE proteins, syntaxin, Munc18, membrane trafficking

## Abstract

Membrane fusion is essential for human health, playing a vital role in processes as diverse as neurotransmission and blood glucose control. Two protein families are key: (1) the Sec1p/Munc18 (SM) and (2) the soluble *N*-ethylmaleimide-sensitive attachment protein receptor (SNARE) proteins. Whilst the essential nature of these proteins is irrefutable, their exact regulatory roles in membrane fusion remain controversial. In particular, whether SM proteins promote and/or inhibit the SNARE-complex formation required for membrane fusion is not resolved. Crystal structures of SM proteins alone and in complex with their cognate SNARE proteins have provided some insight, however, these structures lack the transmembrane spanning regions of the SNARE proteins and may not accurately reflect the native state. Here, we review the literature surrounding the regulatory role of mammalian Munc18 SM proteins required for exocytosis in eukaryotes. Our analysis suggests that the conflicting roles reported for these SM proteins may reflect differences in experimental design. SNARE proteins appear to require C-terminal immobilization or anchoring, for example through a transmembrane domain, to form a functional fusion complex in the presence of Munc18 proteins.

## Abstract 2

Mammalian Munc18 proteins are essential for membrane fusion and human health. Here, we review the literature describing structural and *in vitro* data, and identify a possible explanation for the conflicting functional roles that have been reported.

## 1. Introduction   

Membrane fusion in eukaryotes is essential for many physiological functions, from blood glucose control to neurotransmission. In the secretory and endocytotic pathways, membrane fusion is mediated by the partnership of soluble *N*-ethylmaleimide-sensitive attachment protein receptor (SNARE) proteins and the Sec1p/Munc18 (SM) proteins (Carr & Rizo, 2010 ▶; Südhof & Rothman, 2009 ▶). Mutations in SM proteins are linked with the epileptic conditions Ohtahara syndrome (Saitsu *et al.*, 2008 ▶) and West syndrome (Otsuka *et al.*, 2010 ▶), and knockouts of Munc18a in mice are embryonically lethal (Verhage *et al.*, 2000 ▶). Thus, understanding the molecular basis of membrane fusion mediated by SNARE and SM proteins is highly relevant to health and disease.

**Figure 1:**
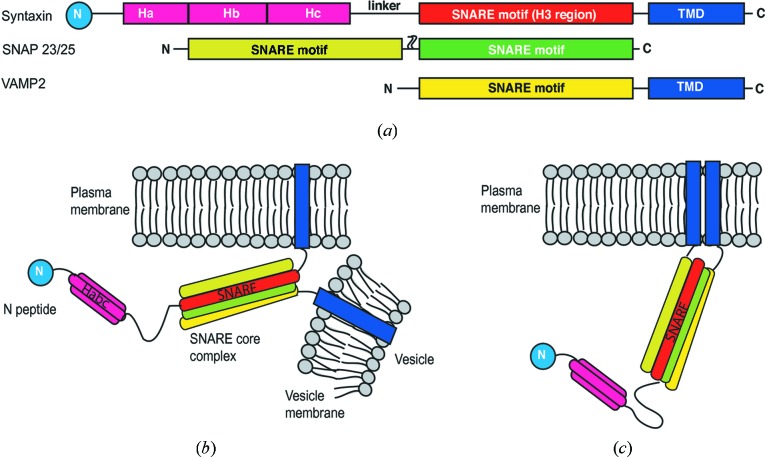
SNARE proteins involved in membrane fusion. (*a*) Domain arrangements of the SNARE proteins: syntaxin, SNAP23/25 and VAMP2 (TMD, transmembrane domain); (*b*) *trans*-SNARE-complex formation through interaction of SNARE motifs on t-SNARE proteins (syntaxin and SNAP on the target membrane) with the SNARE motif of the v-SNARE protein (VAMP2) on the vesicle membrane; (*c*) *cis*-SNARE complex with the TMD of syntaxin and VAMP2 on the same membrane. The blue circle labeled ‘N’ is the N-peptide. Palmitoylation anchors for SNAP23/25 are not shown in panels (*b*) and (*c*).

SNARE proteins fuel fusion events by forming a tight complex across two opposing membranes. SNARE proteins on the target plasma membranes are known as t-SNAREs, whilst SNARE proteins on the vesicle membranes are termed v-SNAREs (Fig. 1 ▶ *a*). In neurons, the SNAREs involved in exocytosis are the t-SNAREs syntaxin 1a (Sx1a) and the synaptosomal-associated protein 25 (or SNAP25), and the v-SNARE vesicle-associated membrane protein 2 (VAMP2 or synaptobrevin). During fusion, t-SNAREs interact with v-SNAREs through their helical SNARE motifs to form a four-helix bundle known as the *trans*-SNARE complex or SNAREpin, that brings the opposing membranes into close proximity for fusion (Fig. 1 ▶ *a*). For a detailed review on the fusion process see the paper by Jahn & Scheller (2006 ▶).

Formation of the *trans*-SNARE complex provides energy to drive the unfavourable bilayer fusion process. Post-fusion, SNARE complexes are termed *cis*-SNARE complexes as they are embedded in the same membrane (Fig. 1 ▶ *c*). The *cis*-SNARE complex is then disassembled by the ATPase *N-*ethylmaleimide-sensitive fusion protein (NSF) and its cofactor soluble NSF attachment protein (SNAP) for recycling (Jahn & Scheller, 2006 ▶).

The SM proteins are known to interact with t-SNARE proteins, such as syntaxin (Sx), and with SNARE complexes (consisting of, for example, Sx, SNAP and VAMP). However, their role in membrane fusion is debated. Here, we highlight the research surrounding the Munc18/Sx interaction for SNARE-complex assembly, and attempt to reconcile the conflicting roles of SM proteins that have been observed. This review will focus on the three mammalian SM proteins (Munc18a, Munc18b and Munc18c, also called Munc18-1, 2, 3) required for exocytotic fusion reactions and their interactions with their highly conserved (>50% identity) Sx binding partners.

**Figure 2:**
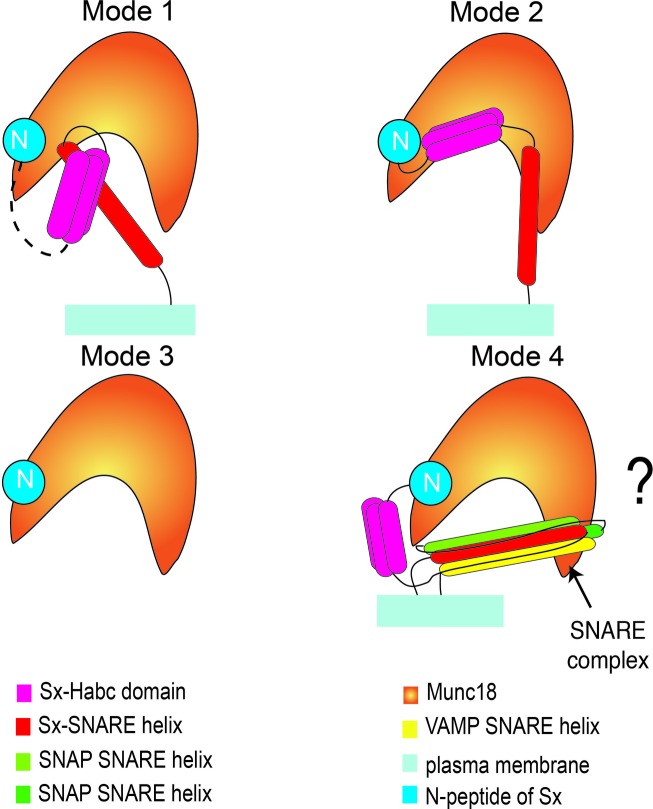
Schematic representation of the proposed binding modes between Munc18 and SNARE proteins. Mode 1: Munc18 binds to a ‘closed’ form of Sx. The N-peptide of Sx binds to a site on Munc18 distinct from the central arched binding cavity. The conformation of the connecting residues between the N-peptide and the H_abc_ domain of Sx in the Munc18/closed Sx-binding mode is unknown (dashed line). Mode 2: Munc18 binds to an ‘open’ form of Sx. Low-resolution solution studies indicate the Munc18 protein binds to an ‘open’ form of Sx, although the details of this binding mode remain to be resolved. Mode 3: Munc18 binds to the N-peptide of Sx. In those proteins where this interaction occurs, the binding mode is highly conserved. Details of this interaction have been defined through crystal structure determination. Mode 4: Munc18 binds to assembled SNARE complex. Although many Munc18 systems have been shown to interact with the pre-assembled SNARE complex (Table 1 ▶), the molecular details of this binding mode are unknown, as indicated by the question mark.

Sxs are modular proteins consisting of a short unstructured N-terminal region (the N-peptide, 1–29 residues) connected to a domain containing three α-helices (H_abc_) linked to a single SNARE helix (H3) followed by a transmembrane domain (TMD) (Fig. 1 ▶ *a*) (Lerman *et al.*, 2000 ▶). The neuronal Sx1a protein, lacking its TMD, was shown *via* NMR to adopt two conformations: a ‘closed’ conformation (where the H_abc_ domain is folded back on its H3 helix preventing it interacting with cognate SNARE partners) and an ‘open’ conformation where H_abc_ is separated from its H3 helix (Dulubova *et al.*, 1999 ▶) (Fig. 2 ▶). However, a recent NMR study on full length Sx1a, including its TMD, reported a higher proportion of the open Sx1a conformation than was observed using the soluble cytosolic domain of Sx1a (Dawidowski & Cafiso, 2013 ▶).

The closed Sx1a conformation, with its SNARE helix sequestered by the H_abc_ domain, is unable to interact with cognate SNARE partners to form the ternary complex required for membrane fusion. Conversely, the SNARE helix of open Sx1a is free to form complexes with partner SNAREs (SNAP25 and VAMP2) and the open Sx1a conformation is thus compatible for fusion. These observations have led to a general hypothesis that a closed-to-open transition of Sxs could represent a switch for controlling membrane fusion.

Although its precise role remains controversial, the importance of SM proteins in disease has been well documented. For example, mutations in Munc18a have been linked with early infantile epileptic encephalopathy (Saitsu *et al.*, 2008 ▶, 2010 ▶) and Munc18b mutations can result in familial hemophagocytic lymphohistiocytosis type 5 (Côte *et al.*, 2009 ▶; Hackmann *et al.*, 2013 ▶). This link between SM proteins and disease has also been confirmed by mutagenesis studies in mice, where deletion of Munc18-1 in mice leads to a complete loss of neurotransmitter secretion from synaptic vesicles throughout development (Verhage *et al.*, 2000 ▶).

SM proteins are conserved from yeast to humans and both yeast genetics and mammalian biochemistry point to a role for SM proteins in regulating SNARE-complex formation. Structural studies on neuronal SM proteins have suggested that Munc18a plays a negative regulatory role in membrane fusion by binding to closed Sx1a, thereby preventing SNARE-complex formation (Burkhardt *et al.*, 2008 ▶, 2011 ▶; Colbert *et al.*, 2013 ▶). However, deletion (Verhage *et al.*, 2000 ▶) or knockdown (Han *et al.*, 2009 ▶) of Munc18a in neurons completely abolishes secretory vesicle fusion and neurotransmitter release, suggesting it plays a positive regulatory role.

Whether SM proteins limit or promote SNARE-complex formation and subsequent membrane fusion may be a consequence of preferential interaction with open or closed Sx, or preferential interaction with *trans*- (pre-fusion) or *cis*- (post-fusion) SNARE complexes (Figs. 1*a* and 1*c*). To date, four interaction modes have been described or proposed as outlined below and summarized in Fig. 2 ▶.

## 2. Binding of SM proteins to SNAREs   

### 2.1. Mode 1: closed Sx   

Crystal structures of three Munc18:Sx systems have been determined: rat Munc18a:Sx1a (Burkhardt *et al.* 2008 ▶), rat Munc18a:Sx1aΔN (Colbert *et al.*, 2013 ▶) and a primordial Unc18:Sx1a complex from *Monosiga brevicollis* (Burkhardt *et al.*, 2011 ▶). These crystal structures show the same closed Sx-binding mode when a soluble C-terminally truncated form of Sx1a was used to generate crystals. In this binding mode, the arch-shaped central cavity of Munc18a accommodates a closed Sx1a conformation (Fig. 2 ▶). Similarly, low-resolution solution scattering models of mouse Munc18a complexed with a soluble C-terminally truncated Sx1a supported a closed binding mode (Colbert *et al.*, 2013 ▶). This interaction, which is not compatible with SNARE-complex formation, points to a role for Munc18a as an inhibitor of membrane fusion (Misura *et al.*, 2000 ▶; Burkhardt *et al.*, 2008 ▶; Chen *et al.*, 2008 ▶; Ma *et al.*, 2011 ▶). However, other studies have drawn another conclusion: that Munc18 binds open Sx.

### 2.2. Mode 2: open Sx   

A hybrid structural biology approach combining X-ray solution scattering, neutron solution scattering, chemical cross-linking, mass spectroscopy and molecular modelling reported that Munc18 binds an open form of Sx (Christie *et al.*, 2012 ▶). Similar to the crystal structures, these experiments used a soluble, C-terminally truncated form of Sx1a or Sx4. For both Munc18a:Sx1a and Munc18c:Sx4 complexes, the low-resolution structural models were consistent with an open conformation of Sx bound to their respective Munc18 proteins (Fig. 2 ▶). On the other hand, when the N-peptide was removed (ΔN-peptide) the binding of the two Sxs to partner Munc18s differed: using the ΔN-peptide/C-terminally truncated Sx constructs the data supported a closed binding mode for Munc18a:Sx1a and no binding for Munc18c:Sx4 (Christie *et al.*, 2012 ▶). This suggests that for Munc18a, the N-peptide regulates the Sx1a-binding mode (closed or open), and therefore affects its regulatory function. For Munc18c, the presence or absence of the Sx4 N-peptide appeared to be an all or none effect, determining whether or not Munc18c interacts with Sx4 (Christie *et al.*, 2012 ▶). The specific binding site for the Sx N-peptide on Munc18 is described in the following section.

### 2.3. Mode 3: N-peptide binding   

A binding site for 10–20 N-terminal residues of Sx has been characterized on some SM proteins (Bracher *et al.*, 2002 ▶; Burkhardt *et al.*, 2011 ▶; Hu *et al.*, 2007 ▶). Whilst this N-peptide-binding mode (Fig. 2 ▶) is not present in all SM proteins, in those where it is present, the binding mode and interactions are highly conserved (Hu *et al.*, 2007 ▶). It is suggested that the N-peptide-binding mode is essential in those SM proteins that act on their own to regulate SNARE assembly, but not those that are part of a large multi-subunit complex – such as the Vps33 family of SM proteins that function as part of a large homotypic fusion and vacuolar protein sorting (HOPS) tethering complex (Baker *et al.*, 2013 ▶).

Crystal structures of SM proteins in complex with their N-peptides have delineated the specificity of this binding mode. Key N-peptide interactions occur between the Asp and Arg side chains on the N-peptide and corresponding oppositely charged regions on domain 1 of the SM proteins. A Leu/Phe from the N-peptide also slots into a hydrophobic pocket on domain 1 of the SM protein. This N-peptide-binding site is distinct from the SM protein central cavity that accommodates the closed binding mode (Christie *et al.*, 2012 ▶; Hu *et al.*, 2011 ▶, 2007 ▶; Latham *et al.*, 2006 ▶; Bracher & Weissenhorn, 2004 ▶).

From examination of the crystal structure of Munc18a in complex with Sx1a and its native N-terminus (PDB code: 4jeu), both the N-peptide and the H_abc_ domain of the same Sx1a molecule could be bound to Munc18a at the same time, even in the closed Sx1a conformation (Colbert *et al.*, 2013 ▶). Residues 10–26 of Sx1a, which immediately follow the N-peptide, could not be modelled in the crystal structure of the Munc18a/Sx1a complex. These 16 residues could potentially stretch from the N-peptide-binding site on Munc18 around to the central arched binding cavity. N-peptide binding to Munc18 has been hypothesized to regulate the closed-to-open transition of Sx, albeit by very different mechanisms (Burkhardt *et al.*, 2008 ▶; Hu *et al.*, 2007 ▶). The possibility that Munc18 can accommodate closed Sx1, whilst simultaneously binding the N-peptide, highlights its potential role in regulating a closed-to-open transition. However, the molecular mechanism by which Sx1a N-peptide binding to Munc18a would induce such a major conformational switch remains a puzzle.

### 2.4. Mode 4: SNARE complex   

SM proteins have also been shown to interact with SNARE proteins in a fourth binding mode whereby Munc18 can bind to the fully assembled SNARE ternary complex (Fig. 2 ▶). This interaction has been demonstrated using both pull-down experiments and liposomal fusion assays (Table 1 ▶) (Dáak *et al.*, 2009 ▶; Latham *et al.*, 2006 ▶; Rodkey *et al.*, 2008 ▶; Shen *et al.*, 2007 ▶). Chemical cross-linking and two-dimensional NMR spectroscopy experiments have confirmed this interaction of Munc18a with an assembled SNARE complex (Dulubova *et al.*, 2007 ▶; Shen *et al.*, 2007 ▶). However, there is no structural data available for this binding mode, and it is unclear whether SM proteins interact with *trans*-SNARE or *cis*-SNARE complexes, or both.

## 3. Experimental techniques used to study SM–SNARE interactions   

A variety of protein–protein interaction techniques have been used to characterize the role of SM and SNARE proteins in membrane-fusion events. Techniques used include: immunoprecipitations, pull-down assays using immobilized protein *via* affinity tags, fluorescence assays, isothermal titration calorimetry assays, surface plasmon resonance kinetics and liposomal fusion assays. These *in vitro* experiments used isolated proteins, either free in solution or immobilized *via* C-terminal or N-terminal affinity tags [glutathione *S*-transferase (GST), or polyhistidine]. Such techniques are used to study whether SM proteins interact with particular Sx constructs.

Alternatively, to study the interaction of soluble proteins with membrane-embedded proteins, *in vitro* liposomal flotation assays or liposomal fluorescent anisotropy experiments can be used. The liposomal fusion assay using fluorescence resonance energy transfer (FRET) to measure lipid mixing is a commonly used approach in the SNARE field, as described by Scott *et al.* (2003 ▶). This is a powerful technique that can measure the rate of fusion between two membranes upon interaction between the protein-binding partners.

Since 1994, many researchers have used these techniques to delineate the role of SM proteins in fusion, though the conclusions of these studies have varied considerably. Table 1 ▶ highlights the conflicting results of SM-protein function studies using different experimental design. Here, we examine a potential link between experimental design and the observed results, in an attempt to reconcile the conflicting conclusions drawn for SM-protein regulation. We propose that the discrepancies reported for the role of Munc18 during fusion could be due to one or more of five causes: the experimental approach taken, N-terminal modification (tags and protease treatment) of the Sx protein, C-terminal anchoring of Sx proteins, the choice of expression system for the proteins to allow post-translational modifications or the presence of lipids in the experiment. We expand on these in turn.

## 4. The effect of experimental technique on SM-protein regulation of fusion   

Experimental design can profoundly affect the results of protein–protein interaction studies. For example, immunoprecipitations and pull-down assays require that the protein is bound to an antibody or constrained in some way. This methodology can sterically hinder protein interactions. Similarly, FRET and fluorescence anisotropy experiments require the addition of a fluorescent probe that can affect interaction between proteins of interest. NMR and protein crystallography provide atomic resolution detail of interaction sites, yet one must be careful with interpretation as these techniques are applied *in vitro* and require complementary mutagenesis studies to confirm the physiological relevance.

Fusion experiments using liposomes present their own problems: even protein-free liposomes can fuse under certain conditions, generating false-positive results (Gad *et al.*, 1979 ▶). Also, this technique relies on the quality and purity of recombinantly expressed proteins. Recently, a new approach measuring vacuole fusion has been reported that may overcome some potential limitations associated with using defined liposomes that are typically composed of simple phosphatidylcholine/phosphatidylethanolamine mixtures (Ko *et al.*, 2014 ▶). In these experiments, the gene encoding Nyv1p, the essential v-SNARE for homotypic vacuole fusion in yeast was deleted to block normal vacuole fusion (Ko *et al.*, 2014 ▶). Vacuoles were isolated from complementary yeast strains, and fusion measured calorimetrically by regeneration of vacuolar phosphatase activity (Ko *et al.*, 2014 ▶). Using this assay, yeast strains were generated that expressed various combinations of neuronal SNAREs and the Munc18a protein, and showed that Munc18a promotes SNARE-mediated membrane fusion of the isolated vacuoles (Ko *et al.*, 2014 ▶). In future, incorporation of various Munc18a and Sx1a mutants may provide new insights into the molecular mechanisms at play during the fusion process.

## 5. The role of the Sx N-peptide   

The presence of an intact Sx N-peptide could also affect the outcome of the experiment. The N-peptide of Sx was absolutely required for Munc18a stimulation of membrane fusion using an *in vitro* liposome fusion assay (whereas the H_abc_ domain was not required) (Rathore *et al.*, 2010 ▶). Removal of an affinity tag from the N-terminus of Sx1a reportedly may remove a few N-terminal residues which, in turn, could reduce the affinity of Sx1a for Munc18a (Burkhardt *et al.*, 2008 ▶) or impact on its binding mode to Munc18a (Christie *et al.*, 2012 ▶). Moreover, Sxs that have been immobilized by their N-termini, or which have the N-terminal tag removed *via* a thrombin protease, could lose their ability to bind Munc18 tightly, or affect their ability to assemble a SNARE complex in the presence of Munc18a (D’Andrea-Merrins *et al.*, 2007 ▶; Rickman *et al.*, 2007 ▶).

On the other hand, an inhibitory role for the N-peptide in SNARE-complex formation was shown by fluorescence spectroscopy experiments for soluble SNARE proteins (Burkhardt *et al.*, 2008 ▶, 2011 ▶). Removal of the Sx1a N-peptide allowed SNARE-complex formation in solution (Burkhardt *et al.*, 2008 ▶). However, in these solution experiments, the Sx1a lacked its transmembrane domain so that the C-terminus was free in solution, which does not reflect the native form of the protein (Table 1 ▶). The effect of C-terminal tethering of Sx is discussed in the next section.

## 6. The effect of the Sx C-terminus   

Native Sxs have a C-terminal TMD anchored into the plasma membrane and connected to the SNARE (H3) helix. Most reported *in vitro* experiments use engineered Sxs with this TMD removed, owing to the difficulty of working with membrane-spanning proteins. Membrane proteins are harder to express, purify and keep stable compared with their soluble protein counterparts.

From examination of the literature, we noted that C-terminal anchoring of Sx may be important for Munc18a to play a positive role in SNARE-fusion regulation (Table 1 ▶). Using an *in vitro* pull-down assay, when Sx1a is immobilized *via* its C-terminus onto affinity beads, Munc18a can assemble the SNARE ternary complex, or bind to an already assembled SNARE ternary complex (Hu *et al.*, 2011 ▶; Malintan *et al.*, 2009 ▶). On the other hand, soluble Sx1a (1-262) that has a C-terminus free in solution was unable to form a SNARE complex in the presence of Munc18a (Burkhardt *et al.*, 2008 ▶) (Table 1 ▶). Similarly, when Sx1a is anchored *via* its C-terminal TMD onto liposomes, Munc18a promotes vesicle fusion in liposomal fusion assays (Rathore *et al.*, 2010 ▶; Rodkey *et al.*, 2008 ▶; Shen *et al.*, 2010 ▶, 2007 ▶; Tareste *et al.*, 2008 ▶) (Table 1 ▶).

For the Munc18b/Sx3 system, when the Sx3 C-terminus is immobilized on affinity beads, Munc18b can bind a preformed cognate SNARE ternary complex (SNAP23/VAMP8/Sx3) (Peng *et al.*, 2010 ▶) (Table 1 ▶). Likewise, Latham *et al.* reported that when Sx4 is immobilized at its C-terminus using affinity beads, it forms a binary complex with Munc18c and can subsequently form a SNARE ternary complex with SNAP23 and VAMP2 (Latham *et al.*, 2006 ▶). This was shown using an *in vitro* pull-down assay and supports the hypothesis that C-terminal anchoring of Sx is important for promoting SNARE-complex formation (Latham *et al.*, 2006 ▶).

**Figure 3:**
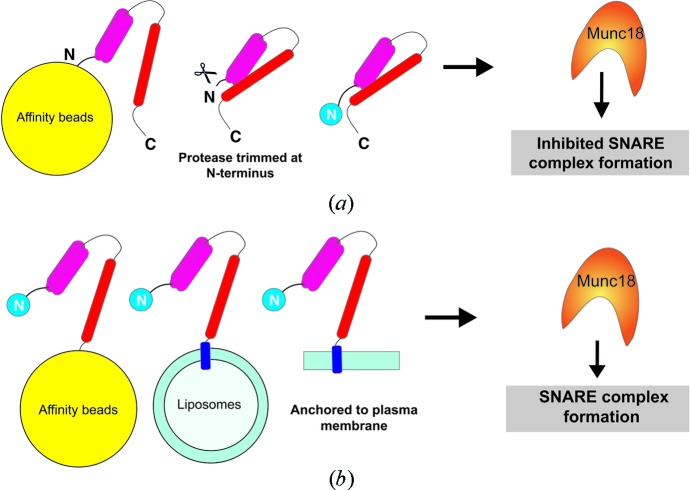
Experimental design and effect on Munc18/SNARE assembly. Different Sx constructs and experimental setups lead to different functional outcomes for Munc18-mediated SNARE-complex formation and membrane fusion. (*a*) Schematic representation of *in vitro* experimental designs used for Sx proteins in solution. Munc18 has been observed to inhibit SNARE-complex formation when the cytosolic domain of Sx was immobilized to affinity beads by the N-terminus, or when the N-terminus is trimmed inadvertently by protease treatment, or when the Sx is free in solution. (*b*) Schematic representation of *in vitro* experimental designs where the Sx constructs were immobilized (affinity beads) or anchored (liposomes/TMD) at their C-terminus. In these experimental setups, SNARE-complex formation was not inhibited in the presence of Munc18. See Table 1 ▶ for more details.

On the other hand, even with the TMD of Sx4 intact, Brandie *et al.* (2008 ▶) observed the opposite result: Munc18c inhibits membrane fusion in a liposomal fusion assay (Table 1 ▶). In these experiments, Brandie *et al.* used an N-terminal His-tagged Sx4 construct and an N-terminal GST-tagged SNAP23 construct. The two t-SNAREs were co-expressed (N-ter-His-Sx4-TMD/GST-SNAP23) and purified using glutathione resin followed by removal of the N-terminal GST-tag from SNAP23 by thrombin cleavage prior to the liposomal fusion assay. As outlined above, thrombin may trim the N-terminal residues of Sx4, and these residues are thought to be critical for binding to Munc18c (Fig. 3 ▶ *a*).

Supporting the hypothesis of the importance of the Sx C-terminus, Yu *et al.* (2013 ▶) showed that Munc18c positively influenced SNARE-complex formation using a liposomal fusion assay similar to that used by Brandie *et al.* (2008 ▶). In these later experiments, the t-SNARE complex consisted of untagged Sx4-TMD and N-terminally His tagged SNAP23 (Yu *et al.*, 2013 ▶) and thrombin was not used.

This assessment of literature *in vitro* experimental data suggests that the N- and C-termini of Sx constructs must closely mimic the native state for Munc18 to positively regulate membrane fusion (Fig. 3 ▶).

## 7. Post-translational modifications   

The use of different expression systems to produce Munc18 proteins might contribute another source of variability. Using a baculovirus expression system rather than a bacterial expression system can potentially introduce modifications to the protein, for example, phosphorylation or glycosylation. Recently we observed that bacterially expressed Munc18c interacts with Sx4 (residues 1-275) with similar affinity to baculovirus-expressed Munc18c, showing that the Munc18c/Sx4 interaction is not dependent on such modifications if they are present (Rehman *et al.*, 2013 ▶). However, whether such modifications are important for Munc18 function in promoting or inhibiting fusion is not well studied and needs to be considered in the future.

## 8. Presence of lipid phosphoinositides   

Phosphoinositides (phosphatidylinositol phospholipids or PtdInsPs) are found on the cytosolic side of cellular membranes and play key regulatory roles in many cellular processes, including membrane trafficking, cell signalling and cytoskeleton organization. Sx1a has been shown to interact and cluster with the predominant form of the plasma membrane phosphoinositide [PtdIns(4,5)P2] through a stretch of polybasic residues (260-KARRKK-265) located adjacent to the TMD of Sx1 (Murray & Tamm, 2009 ▶; van den Bogaart *et al.*, 2011 ▶; Stein *et al.*, 2009 ▶). These polybasic residues are conserved in Sx4 suggesting that interaction with the plasma membrane *via* these residues might also be possible. Supporting this hypothesis, the SNARE and Munc18a mediated lipid-mixing efficiencies of liposomes were shown to be dependent on the presence of PtdIns(4,5)P_2_ (Ma *et al.*, 2013 ▶). The cooperative effect of protein–protein and protein–phosphoinositide interaction in driving protein conformational changes required for membrane trafficking is exemplified by recent studies of the clathrin adaptor AP2 (Kelly *et al.*, 2014 ▶). Whilst the molecular basis for the effect of PtdIns(4,5)P_2_ binding on SNARE-mediated fusion remains unclear, the presence or absence of phosphoinositides in membrane-fusion assays should also be considered in future experimental design.

## 9. Conclusions   

Despite decades of research, the precise regulatory role of SM proteins in membrane fusion remains unclear. Here, we focused on mammalian Munc18 proteins, though Sec1/Munc18 proteins are conserved from yeast through to mammals. Studies in other organisms, such as plants (Park *et al.*, 2012 ▶; Karnik *et al.*, 2013 ▶) and yeast (Peng & Gallwitz, 2002 ▶; Togneri *et al.*, 2006 ▶; Lobingier *et al.*, 2014 ▶) have and continue to contribute to an emerging picture of SM–SNARE function. However, what is obvious from the analysis of mammalian Munc18 proteins is that differing *in vitro* experimental design may contribute to differing outcomes. Removing the Sx TMD and using soluble Sx free in solution often generates different results than when Sx is integrated into a membrane by its C-terminal domain. That is, the anchoring of the Sx C-terminus might be critical to observe SM proteins promoting SNARE-complex formation (Fig. 3 ▶).

Why might anchoring the C-terminus have this effect? We suggest that because the H3 SNARE helix is attached directly to the TMD of Sx (Stein *et al.*, 2009 ▶), removing the TMD somehow alters the conformation of the H3 helix, probably by increasing its flexibility. This change may prevent assembly of Munc18-bound Sx with SNAP and VAMP to form a SNARE four-helix bundle.

Careful consideration is necessary to ensure that engineered Sxs and experimental design of **in vitro** experiments reflect as nearly as possible the native state – that is, C-terminally immobilized Sx with an intact N-peptide. In addition, the same protein constructs should be used throughout, and these should preferentially be the full-length proteins.

A uniform experimental design for studying SM–SNARE interactions is critical so that downstream questions can begin to be addressed. For example, what effect does post-translational modifications of Munc18 and SNARE proteins have on their ability to assemble complexes? Does Sx1a adopt open and closed conformations in the context of the membrane-bound form? Do Sxs other than Sx1a adopt open and closed conformations? What does the crystal structure of Munc18 bound to a full-length membrane-embedded Sx look like? How do SM proteins regulate SNARE-complex formation? Does Munc18 bind a *cis*- or a *trans*-SNARE complex? Do SM proteins and SNARE proteins interact with membrane phospholipids, and if so where do they bind, and what impact does this have on SNARE-complex formation and membrane fusion? Careful consideration must be given in the future to the experimental design and protein constructs used for functional and structural analyses to ensure the field is not further confounded by contradictory results.

## Figures and Tables

**Table 1:** Correlation between experimental design (*i.e.* immobilization of the C-terminus) or Sx construct design (*i.e.* full length or truncated N- or C-terminus) with the reported functional role of Munc18 proteins in *in vitro* SNARE-complex assembly and membrane fusion experiments Sx constructs with a modified N-terminus or that are not C-terminally immobilized are highlighted in **bold**. Papers reporting an inhibitory function for Munc18 are highlighted with ***bold italic***. This summary suggests that Munc18 plays a positive role when using full-length or C-terminally tethered Sx protein constructs in SNARE binding and fusion experiments. Abbreviations: ND not determined in this analysis; TMD transmembrane domain; GST glutathione *S*-transferase fusion tag; His_6_ 6x Histidine fusion tag; Sf9 isolate of *Spodoptera frugiperda* Sf21 cells used for protein production using baculovirus; ITC, isothermal titration calorimetry. The proteins used are mammalian, except for reference *j* where proteins derived from the unicellular choanoflagellate *Monosiga brevicollis* were used.

Experimental information and construct design				Binding of Munc18 to assembled SNARE complex	***Reported function of Munc18 on SNARE-complex formation and membrane fusion, on the basis of *in vitro* experiments***		Ref.†
Sx construct (tag)	Munc18 expression system	Is the Sx N-terminus modified or unmodified?	Is the Sx C-terminus immobilized?	Does Munc18 bind to pre-assembled SNARE ternary complex?	Does Munc18 bound to Sx inhibit or promote SNARE-complex assembly (*i.e.* binding of SNAP and VAMP)?	Does Munc18 inhibit or promote *in vitro* vesicle membrane fusion?	
Sx1a (1-261)	*E. coli*	Unmodified	Yes	Yes	Promote	ND	*a*
(C-ter His)			(Co^2+^ beads)	Munc18a can bind a pre-assembled SNARE complex of Sx1a/SNAP25/VAMP2 in a pull-down assay	Munc18a/Sx1a can bind SNAP25 and VAMP2 in a pull-down assay		
Sx1a (2-265)	*E. coli*	Unmodified	Yes	Yes	ND	ND	*b*
(C-ter His)			(Co^2+^ beads)	Munc18a can bind a pre-assembled SNARE complex of Sx1a/SNAP25/VAMP2 in a pull-down assay			
Sx1a (2-253)	*E. coli*	Unmodified	**No**	Yes	ND	ND	*c*
				Munc18a can bind a pre-assembled SNARE complex of Sx1a/SNAP25/VAMP2 in a gel-filtration shift assay (confirmed by cross-linking and one- and two-dimensional NMR)			
Sx1a (10-253)	*E. coli*	**Modified**	**No**	No	ND	ND	*c*
		**(N-truncated)**		Munc18a does not bind a pre-assembled SNARE complex of Sx1a(10-253)/SNAP25/VAMP2 in a gel-filtration shift assay			
***Sx1a (1-261)***	***E. coli***	Unmodified	**No**	***ND***	***Inhibit***	***ND***	***d***
					***Munc18a/Sx1a does not bind GST-SNAP25 in a GST pull-down assay containing VAMP2***		
***Sx1a (7-261)***	***E. coli***	**Modified**	**No**	***ND***	***Inhibit***	***ND***	***d***
		**(N-truncated)**			***Munc18a/Sx1a(7-261) does not bind GST-SNAP25 in a GST pull-down assay containing VAMP2***		
Sx1a (1-288)	*E. coli*	Unmodified	Yes	Yes	ND	Promote	*e*
			(TMD)	Munc18a binds a pre-assembled t-SNARE complex of Sx1a/SNAP25 in a liposome flotation assay		(Liposome fusion assay)	
Sx1a (1-288)	*E. coli*	Unmodified	Yes	Yes	ND	Promote	*f*
			(TMD)	Munc18a binds a pre-assembled SNARE complex of Sx1a/SNAP25/VAMP2 in a liposome flotation assay		(Liposome fusion assay)	
Sx1a (1-288)	*E. coli*	Unmodified	Yes	ND	ND	Promote	*g*
			(TMD)			(Liposome fusion assay)	
Sx1a (1-288)	*E. coli*	Unmodified	Yes	ND	ND	Promote	*h*
			(TMD)			(Single vesicle FRET assay)	
***Sx1a (1-262)***	***E. coli***	**Modified**	**No**	***ND***	***Inhibit***	***ND***	***i***
***(N-ter His)***		**(His_6_ tag)**			***In solution, Munc18a/Sx1a does not allow an SDS-resistant SNARE complex to form with SNAP25 and fluorescently labelled VAMP2 (confirmed by solution fluorescence anisotropy)***		
Sx1a (25-262)	*E. coli*	**Modified**	**No**	ND	Promote	ND	*i*
(N-ter His)		**(His_6_ tag and truncated)**			In solution, Munc18a/Sx1a(25-262) allows an SDS-resistant SNARE complex to form with SNAP25 and fluorescently labelled VAMP2. No effect on solution fluorescence anisotropy		
***Sx1a (1-279)***	***E. coli***	**Modified**	**No**	***Yes ***	***Inhibit ***	***ND***	***j***
***(N-ter His)***		**(His_6_ tag)**		***Munc18a binds the pre-assembled SNARE complex (*K*_d_ 571nM by ITC)***	***Fluorescence anisotropy in solution***		
Sx1 (20-279)	*E. coli*	**Modified**	**No**	No	No effect	ND	*j*
(N-ter His)		**(His_6_ tag and truncated)**		Munc18a does not bind the pre-assembled SNARE complex (under the conditions used for ITC)	Fluorescence anisotropy in solution		
Sx1a (1-288)	*E. coli*	Unmodified	Yes	ND	ND	Promote	*k*
			(TMD)			(Liposome fusion assay)	
Sx1a (1-288)	*E. coli*	Unmodified	Yes	ND	ND	Promote	*l*
			(TMD)			(Liposome fusion assay)	
***Sx3 (1-260)***	***E. coli***	**Modified**	**No**	***Yes***	***Inhibit***	***ND***	***m***
***(N-ter GST)***		**(GST tag)**		***Munc18b can bind a pre-assembled SNARE complex of SNAP23/VAMP8/Sx3 in a pull-down assay***	***Munc18b/Sx3 does not bind SNAP23/VAMP8 in a pull-down assay***		
Sx3 (28-260)	*E. coli*	**Modified**	**No**	No	Promote	ND	*m*
(N-ter GST)		**(GST tag and truncated)**		Munc18b does not bind a pre-assembled SNARE complex of Sx3/SNAP23/VAMP8 in a pull-down assay	Munc18b/Sx3 binds SNAP23/VAMP8 in a pull-down assay		
Sx4 (1-275)	*Sf9*	Unmodified	Yes	Yes	Promote	ND	*n*
(C-ter His)			(Co^2+^ beads)	Munc18c can bind to a pre-assembled SNARE complex of Sx4/SNAP23/VAMP2 in a pull-down assay	Munc18c/Sx4 binds SNAP23 and VAMP2 in a pull-down assay		
Sx4 (1-298)	*Sf9*	Unmodified	Yes	Yes	Promote	Promote	*o*
			(TMD)	Munc18c can bind to Sx4/SNAP23/VAMP2 in a liposome flotation assay	Munc18c/Sx4 did not inhibit SNARE assembly with SNAP23 and VAMP2 in a liposome flotation assay	(Liposome fusion assay)	
***Sx4 (1-298)***	***E. coli***	**Modified**	***Yes***	***ND***	***ND***	***Inhibit***	***p***
***(N-ter His)***		**(His_6_ tag; thrombin used for GST-SNAP)**	***(TMD)***			***(Liposome fusion assay)***	

† (*a*) Hu *et al.* (2011 ▶), (*b*) Malintan *et al.* (2009 ▶), (*c*) Dulubova *et al.* (2007 ▶), (*d*) Rickman *et al.* (2007 ▶), (*e*) Rodkey *et al.* (2008 ▶), (*f*) Shen *et al.* (2007 ▶), (*g*) Schollmeier *et al.* (2011 ▶), (*h*) Diao *et al.* (2010 ▶), (*i*) Burkhardt *et al.* (2008 ▶), (*j*) Burkhardt *et al.* (2011 ▶), (*k*) Tareste *et al.* (2008 ▶), (*l*) Rathore *et al.* (2010 ▶), (*m*) Peng *et al.* (2010 ▶), (*n*) Latham *et al.* (2006 ▶), (*o*) Yu *et al.* (2013 ▶), (*p*) Brandie *et al.* (2008 ▶).
